# Analysis of the effectiveness of non-pharmaceutical interventions on influenza during the Coronavirus disease 2019 pandemic by time-series forecasting

**DOI:** 10.1186/s12879-023-08640-y

**Published:** 2023-10-24

**Authors:** Hyun Kyung Kim, Kyung-Duk Min, Sung-il Cho

**Affiliations:** 1 Department of Public Health Science, Graduate School of Public Health, Seoul National University, 1 Gwanak-Ro, Gwanak-Gu, Seoul, 08826 Korea; 2 College of Veterinary Medicine, Chungbuk National University, Cheongju, South Korea; 3 Institute of Health and Environment, Seoul National University, 1 Gwanak-Ro, Gwanak-Gu, Seoul, 08826 Korea

**Keywords:** COVID-19, Influenza, Non-pharmaceutical intervention, SARIMA, Social distancing, Time-series forecasting

## Abstract

**Background:**

Coronavirus disease 2019 (COVID-19) was first identified in South Korea during the 2019–2020 seasonal influenza epidemic. The social distancing measures, as effective non-pharmaceutical interventions (NPIs), adopted to mitigate the spread of COVID-19 might have influenced influenza activity. We evaluated IFV(influenza virus) activity during the COVID-19 pandemic and the effect of NPI intensity on influenza transmission.

**Methods:**

IFV activity and epidemic duration during COVID-19 pandemic were predicted under a counterfactual scenario with no NPIs against COVID-19. The Seasonal Autoregressive Integrated Moving Average Model was used to quantify the effects of NPIs on the transmission of influenza virus. Influenza-like illness/1000 outpatients and IFV positivity rate from the 2011–2012 to 2021–2022 seasons were used in this study.

**Results:**

Comparison of the 2020–2021 and 2021–2022 seasonal influenza activities with those in 2013–2019 showed that COVID-19 outbreaks and associated NPIs such as face mask use, school closures, and travel restrictions reduced the influenza incidence by 91%. Without NPIs against COVID-19, the rates of influenza-like illness and IFV positivity would have been high during the influenza epidemic season, as in previous seasons. NPI intensity decreased the transmission of influenza; the magnitude of the reduction increased as the intensity of social-distancing measures increased (weak social distancing; step-by-step daily recovery: 58.10%, strong social distancing; special quarantine measures: 95.12%).

**Conclusions:**

Our results suggest that NPIs and personal hygiene can be used to suppress influenza transmission. NPIs against COVID-19 may be useful strategies for the prevention and control of influenza epidemics.

**Supplementary Information:**

The online version contains supplementary material available at 10.1186/s12879-023-08640-y.

## Introduction

Coronavirus disease 2019 (COVID-19)—declared a pandemic by the World Health Organization (WHO) on March 11, 2020—was first identified in South Korea on January 20, 2020. As of November 3, 2022, more than 635 million people worldwide, and 25 million in South Korea, have developed COVID-19 [1]. Because there was no treatment or vaccine for severe acute respiratory syndrome coronavirus-2 (SARS-CoV-2) in the early stages of the COVID-19 pandemic, non-pharmaceutical interventions (NPIs) were implemented to mitigate its spread. NPIs are actions taken by people and communities to slow the spread of disease [2,3]. For example, in South Korea, individual- and community-level NPIs were implemented in response to the COVID-19 pandemic and a social-distancing policy was established [4–6]. NPIs regarding personal/individual hygiene were also implemented, such as the mandatory use of face masks and ventilation of indoor spaces, use of hand sanitizers, promotion of hand washing and respiratory hygiene, and increased education on public etiquette when coughing/sneezing. Individual-, community-, and government-level NPIs played an important role in controlling COVID-19. Previous modeling studies has shown the effectiveness of NPIs in reducing the spread of SARS-CoV-2 and delaying outbreaks of COVID-19 [7]. The NPIs used to combat COVID-19 significantly altered the patterns and outbreaks of other respiratory diseases, such as adenovirus, parainfluenza virus, metapneumovirus, and influenza virus (IFV).

**Fig. 1:**
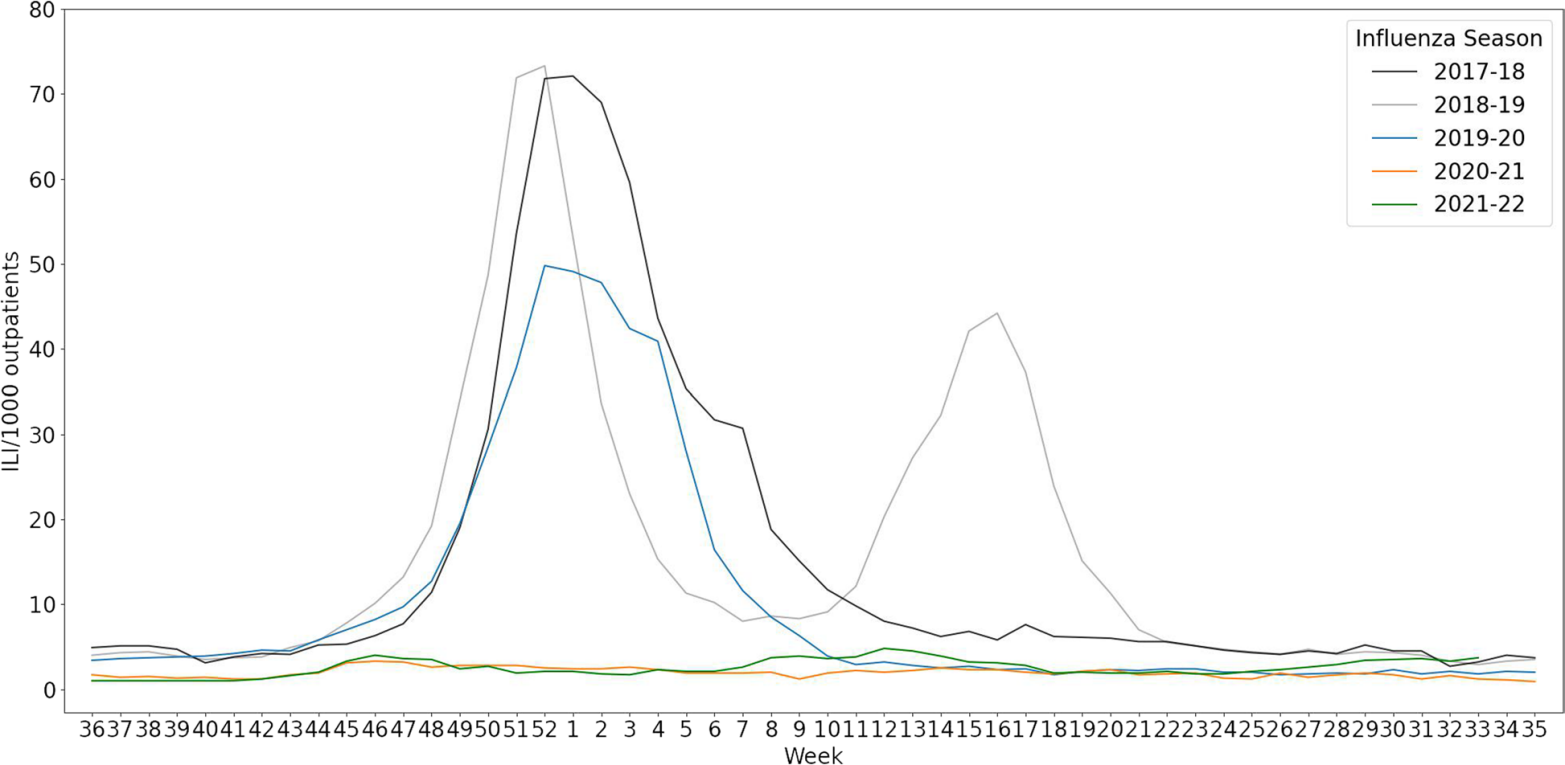
Trends of influenza-like illnesses (ILIs) in the 2017–2018 to 2021–2022 seasons

During the COVID-19 pandemic, the incidence and rate of hospitalization for influenza infection decreased, the circulating virus strains changed, and the seasonality of influenza infection was disrupted despite a consistent level of influenza vaccination coverage in South Korea [8–10]. Among IFV B lineages, Yamagata has not been detected since March 2020 and other lineages have exhibited less genetic diversity compared to previous seasons [11, 12]. The detection rates of IFV, parainfluenza virus, and metapneumovirus decreased markedly beginning in week 13 of 2020 [13]. Also, the incidence pattern of influenza changed considerably, including its seasonality. The number of influenza-like illnesses (ILIs) per 1000 outpatients decreased during the COVID-19 pandemic compared to previous influenza seasons (Fig. 1). During the 2020–2021 season, no influenza advisory was issued, for the first time since the 2000–2001 season when the first flu advisory was issued by the Korea Disease Control and Prevention Agency [14].

Influenza and COVID-19 have similar symptoms and transmission routes [15–17]. The effectiveness of NPIs in mitigating the spread of viruses differ according to the transmissibility, latent period, and serial interval of the virus in question [18]. The effects of quarantine policies are maximized when the latent period is shorter than the incubation period. Also, the effects of NPIs are maximized for diseases with short durations of infectiousness [18]. Compared to SARS-CoV-2, IFV has a short serial interval and its viral excretion peaks early [19–21]. These features enable the rapid spread of IFV, which could reduce the effects of quarantine and isolation measures on its spread. As such, it has been proposed that NPIs would not be effective in controlling influenza outbreaks [22]. However, few studies have investigated the effects of NPIs on IFV transmission due to the high socioeconomic cost that hampers research on the effects of NPIs. Because NPIs were implemented to control COVID-19, research on the effects of NPIs on non-COVID-19 diseases is needed to formulate guidelines on infection prevention and control.

Concern over waning immunity of influenza has been raised because population-level immunity to IFV results from prior infection and vaccination [10]. The circulation of IFV was low in the prior 2 years, possibly reducing population-level immunity. This can make selection of vaccine strains problematic and potentially reduce vaccine effectiveness, because strain selection is based on previous hemagglutinin inhibition antibody titers against circulating IFV strains from the Northern and Southern Hemispheres [10].

We evaluated influenza outbreak patterns during the COVID-19 pandemic and the effects of NPIs on influenza activity by predicting ILIs/1000 outpatients, IFV positivity rate and epidemic duration under a counterfactual scenario with no NPIs against COVID-19 using time-series forecasting. The predicted value was compared to the observed value during COVID-19 pandemic. The findings provide insight into the effects of NPIs on influenza.

## Methods

### Data sources

The Korea Influenza and Respiratory Viruses Surveillance System was established by the Korea Disease Control and Prevention Agency (KDCA) to monitor changes in the pattern and incidence of IFV. In cooperation with medical institutions, the KDCA reports the results of respiratory virus surveillance,including IFV, and performs genetic analyses to determine the causes of outbreaks and monitor the emergence of new and antiviral-resistant IFVs [23]. Based on this surveillance system, the KDCA issues and lifts influenza advisories in accordance with the annual ILI baseline.

ILI data obtained from the KDCA infectious disease website (https://www.kdca.go.kr/npt/) were used to evaluate influenza activity during the COVID-19 pandemic and predict influenza cases under the counterfactual scenario (no NPIs against COVID-19) [24]. ILI was defined as number of cases with sudden fever > 38℃ and cough or sore throat. The ILIs/1000 outpatients are the rate of ILIs among the total number of weekly outpatients, which is reported weekly by the KDCA. ILI data from the 2011–2012 to 2021–2022 seasons were used in this study.

Laboratory respiratory virus surveillance data are publicly available via the Pathogens & Vector Surveillance Weekly Report of the KDCA (https://www.kdca.go.kr/npt/) and on the FluNet website (https://www.who.int/tools/flunet/). For IFV, the numbers of positive samples for IFV A (H1N1/pdm09), IFV A (H3N2), IFV B (Victoria lineage) and IFV B (Yamagata lineage) were assessed (Figure S1). The IFV B positivity rate was not analyzed because the IFV B (Yamagata lineage) was not detected during the COVID-19 pandemic [25].

Information on the social-distancing level in South Korea was confirmed in the press releases of KDCA and the Ministry of Health and Welfare. Social-distancing level refer to tiered system of guideline and restrictions implemented by the government to control and mage the spread of COVID-19. These levels are designed to provide a structured and adaptable approach to public health measures based on the severity of the situation. The social-distancing intensity was categorized into four levels (Very strong, Strong, Moderate and Weak) based on the level of social distancing implemented in South Korea. These levels were determined according to the specific NPIs that were implemented (Table S1).

### Descriptive analysis

Indicators of influenza activity—ILIs/1000 outpatients, number of IFVs detected, and IFV positivity rate—by social distancing timing and intensity were analyzed to assess the IFV activity and change of incidence pattern from the 2013–2014 to 2021–2022 seasons.

### Time-series analysis

**Fig. 2:**
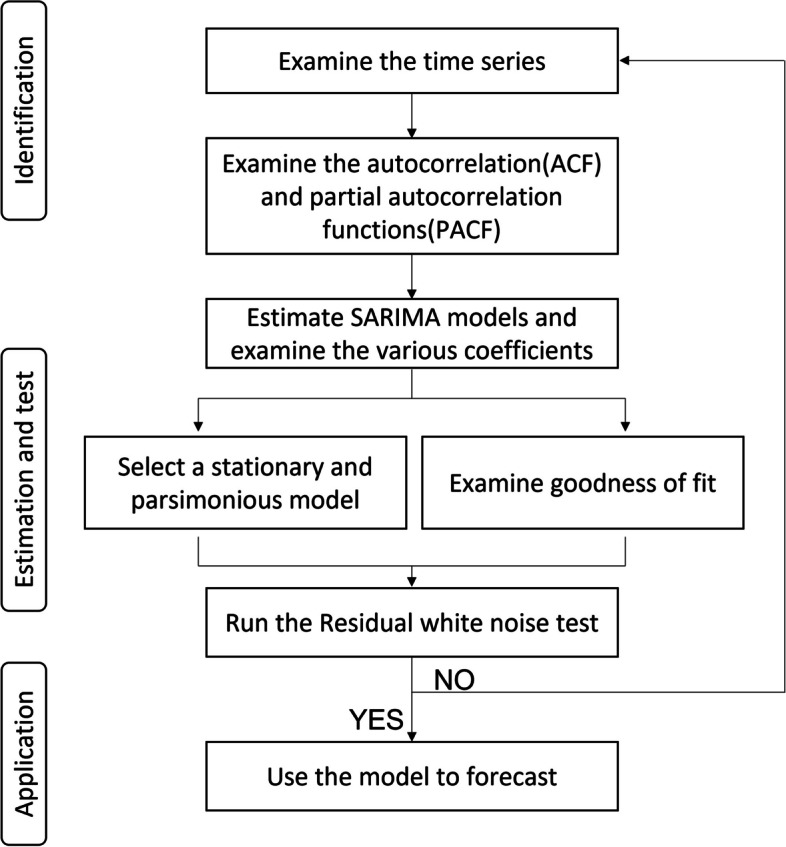
Flow diagram of SARIMA forecasting

Because of the strong seasonality of influenza data, the Seasonal Autoregressive Integrated Moving Average (SARIMA) model was used to forecast influenza activity and the IFV virological trend. The generalized Box–Jenkins time-series forecasting method has four steps: identification, estimation, diagnostic checking, and forecasting (Fig. 2). Before forecasting, stationarity was assessed using the KPSS and ADF tests and classical additive decomposition was conducted to identify the trend, seasonality, cycle, and random variation of the time series [26]. The decomposition results suggested that the ILIs/1000 outpatients, IFV positivity rate, and IFV A positivity rate showed strong seasonality and irregular trends. The autocorrelation function and partial autocorrelation function were tested to analyze the characteristics of the time-series data and identify an order appropriate for the SARIMA forecasting model. The Akaike’s information criterion with correction for small sample size(AICc) values were utilized to identify the each components in the SARIMA model. The components were chosen based on the smallest AICc values. Using the selected model, the number of ILIs/1000 outpatients, IFV positivity rate, and IFV A positivity rate were estimated under the counterfactual scenario. The Ljung–Box test was conducted to check the residual; if the residual was white noise, then forecasting was carried out by fitting the time-series data into the selected SARIMA model. To assess the accuracy of model predictions, we utilized the data from the 2011–2019 season to make forecasts regarding influenza activity following COVID-19. The accuracy of forecasts was determined by calculating the mean absolute error(MAE), mean absolute scaled error(MASE), root mean square scaled error(RMSSE) and mean absolute percentage error(MAPE). Upon analyzing the variance between observed and predicted values, we observed that the selected SARIMA models exhibited strong predictive performance for the following categories: ILIs/1000 outpatients (mean absolute scaled error: 0.302), IFV positivity rate (MASE: 0.308), and IFV A positivity rate (MASE: 0.390) (Table S5).

## Results

### Influenza activity in South Korea

**Fig. 3:**
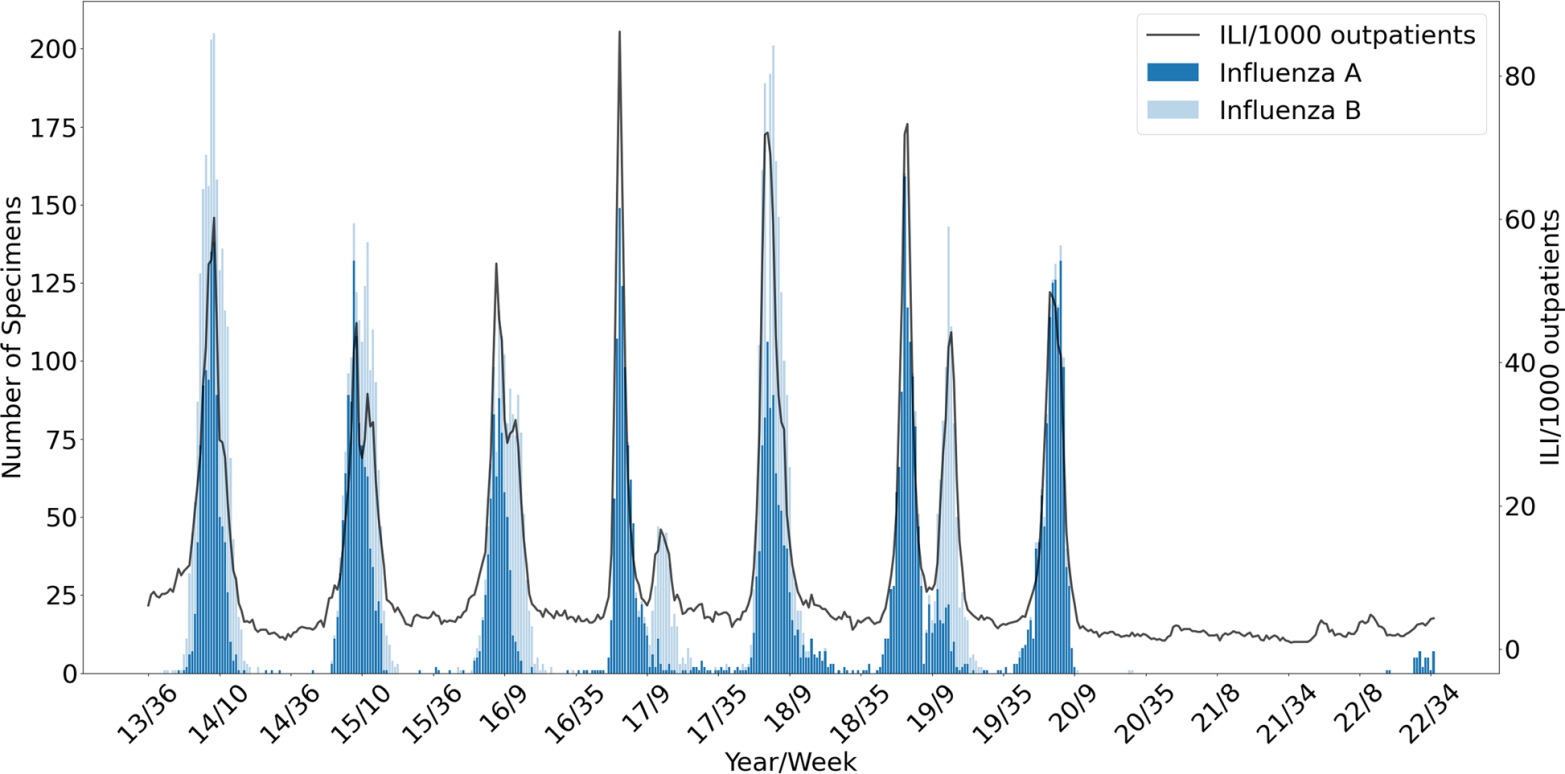
Influenza-like illness (ILI) and influenza virus laboratory surveillance in South Korea, 2013–2022

During the 2020–2021 and 2021–2022 seasons, the influenza epidemic duration decreased, and no influenza advisory was issued, unlike in other seasons (Table S2). During the COVID-19 pandemic, the average weekly number of IFV samples decreased from 215 (2013–2020 seasons) to 97; the IFV positivity rate also decreased (2013–2020 seasons, 14.33%; 2020–2021 season, 0.00%; 2021–2022 season, 0.64%) (Table 1). From the 2013–2014 to 2021–2022 seasons, we observed three types of epidemic patterns: a unimodal distribution with co-circulation of IFV A and IFV B, a bimodal distribution of IFV A and IFV B, and predominance of IFV A throughout the influenza epidemic period (Fig. 3). The influenza season showed a unimodal pattern (2013–2014, 2015–2016, and 2017–2018 seasons) with one large peak of co-circulation of IFV A and B. The 2016–2017 and 2018–2019 seasons both showed a bimodal pattern, in which IFV A predominated during the first peak and IFV B during the second peak. During the 2019–2020 season, when COVID-19 emerged, IFV A predominated (positivity rate, 95.81%); by contrast, IFV B was rarely detected (Table 1).

**Table 1:** Influenza virus laboratory surveillance data

Season^a^	Total specimens	Number of detected specimen (positivity rate %)			IFV total
		IFV A H1N1(pdm09)	IFV A H3N2	IFV B	
2011–2012	14,628	1 (0.00%)	1950 (51.5%)	1834 (48.5%)	3785 (25.88%)
2012–2013	13,951	332 (19.48%)	1276 (74.88%)	96 (5.63%)	1704 (12.21%)
2013–2014	12,343	346 (16.52%)	640 (30.56%)	1108 (52.91%)	2094 (16.97%)
2014–2015	11,065	176 (10.94%)	836 (51.96)	597 (37.10%)	1609 (14.54%)
2015–2016	10,933	582 (44.09%)	62 (4.70%)	675 (51.14%)	1320 (12.07)
2016–2017	11,526	6 (0.50%)	882 (72.89%)	322 (26.61%)	1210 (10.50%)
2017–2018	11,989	141 (7.00%)	771 (38.30%)	1101 (54.69%)	2013 (16.79%)
2018–2019	11,862	760 (41.90%)	379 (20.89%)	675 (37.21%)	1814 (15.29%)
2019–2020	8640	825 (70.45%)	297 (25.36%)	49 (4.18%)	1171 (13.55%)
2020–2021	4334	0 (0.00%)	0 (0.00%)	0 (0.00%)	0 (0.00%)
2021–2022	5959	0 (0.00%)	38 (100.00%)	0 (0.00%)	38 (0.64%)

^a^Influenza season defined as week 36 to week 35 of the following year

In the 2019–2020 season, the ILIs/1000 outpatients gradually decreased after a peak at week 52. The ILIs/1000 outpatients from weeks 1 to 8 of 2020 was higher than that in the same week in 2019 (Figure S2). However, it decreased rapidly after week 4, when the first COVID-19 case was confirmed in South Korea. After week 13 (when social distancing was implemented), the ILIs/1000 outpatients remained < 3 until the end of the season.

In the first 3 weeks of 2020, the ILIs/1000 outpatients and IFV positivity rates increased 55.04% and 27.68% respectively from the reference year (2014–2019). The mean difference was 16.48 ILIs/1000 outpatients, which was not significant after adjusting for the effect of week (Table 2). The first COVID-19 case was confirmed in week 4 and from weeks 4 to 10, the ILIs/1000 outpatients and IFV positivity rates decreased significantly to 38.23% and 49.49%, respectively. After the WHO pandemic declaration in week 11, the South Korean government implemented enhanced social distancing from weeks 13 to 16, which reduced the ILIs/1000 outpatients and IFV positivity rates by 86.78% and 100%, respectively.

**Table 2:** Influenza incidence in the 2020 and 2014–2019 seasons by social-distancing period

Week no	W1 ~ W3	W4 ~ W10	W11 ~ 16	W17 ~ 33	W34 ~ 41	W42 ~ 47	W48 ~ 52
	20.1.1 ~ 1.18	1.19 ~ 2.7	2.8 ~ 2.18	2.19 ~ 8.15	8.16 ~ 10.10	10.11 ~ 11.21	11.22 ~ 12.31
	Before COVID-19	Alert level red	Pandemic declared, Enhanced SD	Relaxed, Daily life, SD 1	SD 2, 2.5	SD 1	SD 2, 2.5, 3
ILI/1000 outpatients							
2020							
Mean	46.43	16.51	2.73	2.04	1.58	2.40	2.67
Median	47.80	11.60	2.75	2.00	1.45	2.50	2.70
IQR	3.35	14.80	0.33	0.50	0.40	1.43	0.28
2014–2019							
Mean	29.95	26.74	20.68	5.72	3.82	5.38	29.67
Median	23.05	27.90	19.40	4.70	3.85	4.55	19.20
IQR	23.20	31.05	17.68	1.78	0.90	2.00	41.00
Percent reduction	-55.04	38.23	86.78	64.32	58.74	55.40	91.01
Mean difference	16.48	-10.22	-17.94	-3.68	-2.24	-2.98	-25.57
*p*-value	0.206	0.132	< 0.005	< 0.005	< 0.005	< 0.005	< 0.05
Number of detected IFV All (A/H1N1, A/H3N2, B)							
2020							
Mean	125.00	46.29	0.00	0.12	0.00	0.00	0.00
Median	126.00	35.00	0.00	0.00	0.00	0.00	0.00
IQR	6.50	65.50	0.00	0.00	0.00	0.00	0.00
2014–2019							
Mean	83.22	85.98	68.58	6.22	0.90	6.17	60.32
Median	79.00	92.50	67.00	2.00	0.50	2.00	50.00
IQR	55.00	94.50	57.25	5.00	1.00	8.00	99.50
Percent reduction	-50.20	46.16	100.00	98.10	100.00	100.00	100.00
Mean difference	41.78	-39.69	-68.58	-6.10	-0.90	-6.17	-56.72
*p*-value	0.204	0.101	< 0.005	< 0.005	< 0.005	0.117	< 0.05
IFV positivity rate							
2020							
Mean	40.53	17.58	0.00	0.16	0.00	0.00	0.00
Median	40.56	15.84	0.00	0.00	0.00	0.00	0.00
IQR	2.93	26.16	0.00	0.00	0.00	0.00	0.00
2014–2019							
Mean	31.74	34.81	26.18	2.69	0.45	2.40	20.81
Median	31.14	39.02	26.89	1.00	0.21	0.83	16.67
IQR	21.72	38.34	22.10	2.59	0.61	3.08	32.26
Percent reduction	-27.68	49.49	100.00	94.13	99.78	99.96	100.00
Mean difference	8.79	-17.23	-26.18	-2.53	-0.44	-2.40	-19.67
*p*-value	0.390	0.0566	< 0.005	< 0.005	0.0511	0.0981	< 0.05

^a^*ILI* Influenza-like illness, *IFV* Influenza virus, *IFV* Positivity rate, number of detected IFVs /total specimens, *IQR* Interquartile range, *SD* Social distancing

Percentage reduction: (mean of 2014–2019 − mean of 2020) ÷ (mean of 2014–2019) × 100

^b^W1–3: before COVID-19, W4–10: alert level red, W11–16: pandemic declared, SD, W16–33: relaxed SD, distancing in daily life, Level 1 SD, W34–41: Level 2 SD, W42–47: Level 1, 1.5 SD, W48–52: Level 2, 2 + a, 2.5 SD

^c^Detailed summary of social distancing (Table S1)

Implementation of social distancing in daily life (weeks 17–33) considerably reduced the ILIs/1000 outpatients and IFV positivity rates. However, the mean difference was small because the period in question was not within the influenza epidemic season (mean difference, ILI − 3.68 [*p* < 0.05], IFV − 2.5343 [*p* < 0.05]). From weeks 48 to 52 of 2020, when very strong social distancing was implemented, the ILIs/1000 outpatients and IFV positivity rates decreased significantly. This period was within the influenza epidemic period when there was no COVID-19.

### Time-series forecasting

**Fig. 4:**
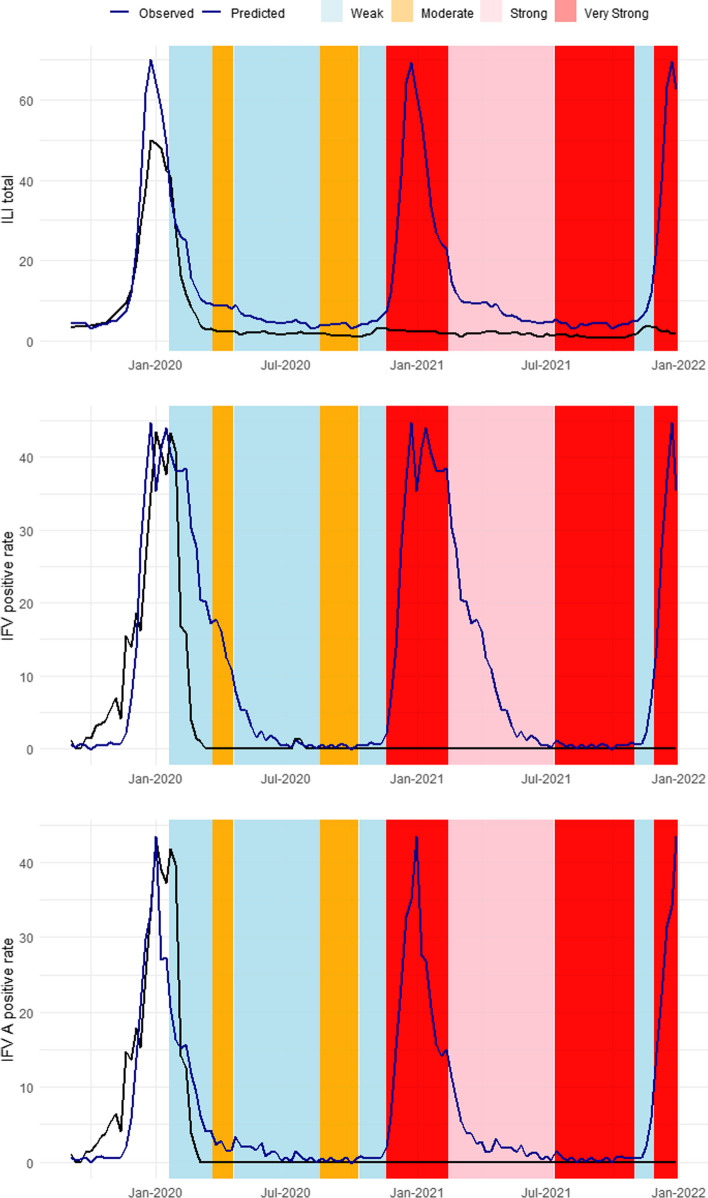
Forecasting results (week 35 of 2019 to week 52 of 2021). * Black line, observed influenza-like illness (ILI); blue line, forecasted ILI. ** A timeline of social distancing in South Korea is provided in Table S1. **a** Forecasts of ILIs/1000 outpatients. **b** Forecasts of the influenza virus (IFV) positivity rate. **c** Forecasts of the IFV A positivity rate

SARIMA models were established to fit the 2011–2019 seasons and predict influenza activity during the COVID-19 pandemic under the counterfactual scenario (Table S3, Figure S3). Significant differences were observed between the predicted and observed values during the period of implementation of NPIs against COVID-19, and the differences varied according to the level of social distancing (Fig. 4). Under the counterfactual scenario, the ILIs/1000 outpatients, IFV positivity rate, and IFV A positivity rate showed trends similar to those of the 2011–2019 seasons. From week 5 of 2020—the week after COVID-19 emergence—to the end of 2020, the ILIs/1000 outpatients, IFV positivity rate, and IFV A positivity rate decreased by 71.80%, 73.94%, and 83.33%, respectively. In 2021, the ILIs/1000 outpatients decreased by 87.12% compared to the predicted value under the counterfactual scenario and the IFV and IFV A positivity rates decreased by 99.9% (Table 3).

**Table 3:** Observed and predicted influenza-like illness incidence under the counterfactual scenario

	Before COVID-19		After COVID-19					
	2014–2019	2019–2020 (2019 W36 ~ 2020 W4)	2020 (2020 W51 ~ 2020 W52)			2021		
	Observed^a^	Observed	Observed	Predicted^b^	% Change	Observed	Predicted	% Change
ILI total	13.91	16.11	3.45	12.22	-71.80	1.90	14.78	-87.12
Positive rate of IFV (%)	12.92	14.03	1.68	10.09	-73.94	0.0014	11.93	-99.99
Positive rate of IFV A (%)	7.60	7.21	1.47	5.63	-83.33	0.0014	6.95	-99.98

2014–2019: week 36 of 2014 to week 35 of 2019

2019: week 1 of 2019 to week 52 of 2019

2020: week 5 of 2020 to week 52 of 2020

Week 5 of 2020: The first week after COVID-19 emergence

2021: week 1 of 2021 to week 52 of 2021

Percentage change = 100 × (observed- predicted)/predicted

*IFV* Influenza virus, *IFV A* Influenza virus A (H1N1/pdm09 and H3N2)

^a^Observed value is the average value of the base period

^b^Predicted under the counterfactual scenario (no non-pharmaceutical intervention)

### Forecasting influenza epidemic duration and peaks

The duration of flu epidemics and peak points are estimated based on the forecasting results of ILIs/1000 outpatients (Table 4). The epidemic duration defined as the number of weeks between the week when the flu advisory is issued and the week when it is lifted, based on the ILI baseline. The ILI baseline for the 2019–2020 season was 5.9, and the epidemic lasted for 20 weeks (from week 46 of 2019 to week 13 of 2020). However, under the counterfactual scenario, the epidemic is expected to end in week 25 with a peak of 64.06 ILIs/1000 outpatients in week 1. There were no flu epidemics in the 2020–2021 and 2021–2022 seasons in the real world. However, under the counterfactual scenario, the duration of epidemics is expected to be 31 weeks for both the 2020–2021 and 2021–2022 seasons. The estimated peak points are 64.33 in week 51 of the 2020–2021 season and 69.37 in week 52 of the 2021–2022 season. These findings are similar to the previous seasons before the COVID-19 pandemic.

**Table 4:** Predicted influenza epidemic duration and peak values

	Season	ILI baseline	Duration of Epidemics	Peak ILIs/1000 outpatients(week)
observed	2018–2019	6.3	32 weeks (W46 ~ W25)	73.3(W52)
	2019–2020	5.9	20 weeks (W46 ~ W13)	49.8(W52)
predicted	2019–2020	5.9	32 weeks (W46 ~ W25)	64.06(W1)
	2020–2021	5.8	31 weeks (W47 ~ W25)	64.33(W51)
	2021–2022	5.8	31 weeks (W46 ~ W24)	69.37(W52)

### Effects of NPIs on ILI

The greatest percentage difference of ILIs/1000 outpatients between the predicted and observed values in 2020–2021 occurred from weeks 48 to 52 in 2021 and from weeks 48 to 6 in the 2020–2021 season (Table 5). From week 48 of 2020 to week 6 of 2021, the observed ILIs/1000 outpatients decreased by 93.83% under the counterfactual scenario compared to the predicted value. During this period, very strong NPIs were implemented (social distancing levels 2 and 2.5). In addition, from weeks 48 to 52 of 2021—when social distancing was strengthened compared to the previous season— the ILIs/1000 outpatients decreased by 95.12%. By contrast, the percent change was low during periods of easement of social-distancing measures. For example, during the period of weak NPI were implemented (social-distancing level 1, weeks 42–47 of 2020;step-by step daily recovery, weeks 44–47 of 2021), the observed ILIs/1000 outpatients decreased by 55.06% and 58.10%, respectively, compared to the forecasted values. Therefore, the intensity and timing of NPIs influenced influenza transmission.

**Table 5:** Observed and predicted influenza-like illness values under the counterfactual scenario by non-pharmaceutical period

		2020						2021			
	Week no^a,^^b^	W4–10	W11–16	W17–33	W34–41	W42–47	W48–6	W7–27	W28–43	W44–47	W48–52
		20.1.19–2.7	2.8–2.18	2.19–8.15	8.16–10.10	10.11–11.21	20.11.22–21.2.13	2.14–7.10	7.11–10.30	10.31– 11.27	11.28- 12.31
ILI observed	Mean	16.5	2.7	2.0	1.6	2.4	2.5	1.9	1.3	3.2	2.5
	Median	11.6	2.8	2.0	1.5	2.5	2.5	1.9	1.2	3.5	2.4
	IQR	14.8	0.3	0.5	0.4	1.4	0.3	0.4	0.6	0.7	0.6
ILI predicted	Mean	22.2	8.9	5.3	4.1	5.3	39.9	7.8	4.2	7.7	51.7
	Median	24.9	8.9	4.8	4.3	4.9	37.1	7.3	4.4	6.9	62.5
	IQR	13.2	0.4	1.4	0.4	1.4	31.7	4.6	0.5	2.8	23.4
ILI % change^c^		-25.55	-69.34	-61.18	-61.34	-55.06	-93.83	-75.66	-70.23	-58.10	-95.12
Average COVID-19 case^d^		1108	490	316	1103	1132	4439	4141	12,419	20,424	39,460

^a^W1–3: Before COVID-19, W4–10: first case confirmed, alert level red, W11–16: pandemic declared, social distancing (SD), W16–33: relaxed SD, distancing in daily life, Level 1 SD, W34–41: Level 2 SD, W42–47: Level 1,1.5 SD, W48–6: Level 2, 2 + a, 2.5 SD

W7–27: Level 2 SD, W28–43: Level 4 SD, W44–47: Step-by-step daily recovery, W48–52: Special quarantine measures

^b^A detailed summary of social distancing is provided in Table S1

^c^Influenza-like illness (ILI) percentage change = 100 × (observed- predicted)/predicted

^d^Average COVID-19 cases: Average number of newly confirmed COVID-19 cases in South Korea

## Discussion

Decreases in influenza activity were associated with the intensity and timing of NPIs against COVID-19. The positivity rates of IFV and its subtypes were considerably lower during the COVID-19 pandemic compared to previous seasons. During the 2020–2021 season, IFV was not detected and no IFV B lineage was detected after the emergence of SARS-CoV-2 in South Korea. This has been replicated elsewhere; Nextstrain and FluNet last reported IFV B/Yamagata lineage in March 2020 [10, 27]. Compared to the 2013–2014 to 2018–2019 seasons, the IFV and IFV A positivity rates decreased by 58.11% and 99.02%, respectively. Accordingly, the 2019–2020 season showed a different virological pattern, with the lowest-ever IFV B positivity rate. The virological pattern can be divided into co-circulation of IFV A and B throughout the influenza epidemic or IFV A predominance followed by IFV B predominance during the second peak. The early stage of the COVID-19 pandemic (*i*.*e*., week ≥ 9) corresponds to the typical influenza epidemic. The NPIs against COVID-19 may have inhibited the emergence and spread of IFV B in the community. School closures could also explain the unusual pattern of IFV B, because IFV B circulates more actively among children than among adults [28–30]. During the COVID-19 pandemic, no influenza advisory was issued and after the emergence of COVID-19, the peak number of ILI cases was smaller than in previous seasons (72.1 and 73.3 ILIs/1000 outpatients in the 2017–2018 and 2018–2019 seasons, respectively). This might be a result of the early lifting of influenza advisories after the 2019–2020 season.

The IFV positivity rate and the number of ILIs/1000 outpatients in 2020 differed from those in previous seasons. The greatest reduction of ILIs/1000 outpatients occurred from weeks 48 to 52—when level-2 social distancing was implemented, and private gatherings of five or more people were prohibited—and from weeks 11 to 16—when enhanced social distancing was implemented (weeks 12–16) after the pandemic declaration in week 11.

The forecasted values suggest that the NPIs reduced ILIs/1000 outpatients by 71.80% in 2020 and 87.12% in 2021. The influenza epidemic duration and peak timing were also similar in these years. The percentage difference between the observed and predicted ILIs/1000 outpatients under the counterfactual scenario was greatest during periods when social distancing was strongest. Therefore, the timing and intensity of NPIs affected influenza activity.

Our results suggest that NPIs against COVID-19 (*e*.*g*., hand hygiene, mask wearing, respiratory etiquette, travel restrictions, and staying at home with respiratory symptoms) reduced influenza activity [16, 31–36]. IFV has an incubation period of 2 days; therefore, an overseas entrant infected with IFV may not spread the virus to others because of the 14-days mandatory quarantine. Indeed, travel restrictions reportedly delay influenza transmission and alter the timing of epidemic peaks by delaying spread by 2–19 weeks [37].

This study has several limitations. First, the decrease in ILI cases might be a result of fewer visits to medical institutions, thereby potentially leading to the under-reporting of influenza activity. The policy directing individuals with respiratory symptoms to specialized clinics may have contributed to fewer outpatient visits and an underestimation of actual IFV infections. Additionally, the inclination of individuals with mild symptoms or chronic conditions to avoid medical facilities could further contribute to under-reporting. However, the ILI cases per 1000 outpatients metric, while not directly indicative of a reduction in total patient numbers seeking care, offers a relative perspective on ILI prevalence within a population. This approach helps to address these limitations to some extent. Second, the laboratory respiratory surveillance data do not represent the total number of IFV cases in South Korea. Because only samples from patients who visited designated institutions were tested, unconfirmed influenza infections might have been missed. Third, the high MAPE of positive rate of IFV and positive rate of IFV A observed in the forecasting results. This high MAPE is primarily attributed to the nature of the datasets utilized in this study, which consist of proportional data and include instances of zero values. When forecasting proportional data with zero values, models face discontinuities in the data, as zero values represent periods when no cases were reported. To mitigate the impact of zero values and enhance the accuracy of our assessment, we focused on calculating MAPE during the peak season. The peak season was defined as the period during which the positivity rate exceeds 10%, and during this period, the MAPE value demonstrated higher accuracy. Finally, the SARIMA forecasting model did not consider the IFV B positivity rate. Also, the scenario did not consider the possibility of the emergence of a new influenza subtype. Regardless, assuming that the pattern of influenza activity is maintained, the forecasting model is appropriate.

Influenza imposes a considerable socioeconomic burden as a result of its high rates of mortality and morbidity. In South Korea, the rate of mortality from influenza is high among people ≥ 65 years of age compared to other age groups [38, 39]. Concern regarding dual epidemics of influenza and COVID-19 has been raised; therefore, a rapid public-health response to influenza is important. In this study, the number of ILI cases and the IFV and IFV A positivity rates were lower than the predicted values in 2020 and 2021 under the counterfactual scenario. This suggests the efficacy of social-distancing and public-health measures such as face mask use, hand washing, school closures, and travel restrictions. The intensity and timing of NPIs were linked to changes in influenza transmission. Early detection of influenza epidemics enables preparations to be made before an influenza advisory is issued, reducing the burden of illness. Also, influenza vaccination and personal hygiene can be promoted prior to the start of an influenza epidemic.

Immunity may have changed during the COVID-19 pandemic because of reduced IFV circulation and a lower vaccination rate. In addition, vaccine strain selection is hampered by the detection of the relatively small number of IFV subtypes in the past 2 years. These issues emphasize the importance of research on the effects of public-health measures on influenza transmission. Our findings provide insight into the effects of COVID-19-targeted NPIs on influenza. However, because some NPIs are socioeconomically costly, their effectiveness needs to be evaluated further.

## Conclusion

NPIs targeted at COVID-19 affected the transmission of IFV. Social distancing, which reduced SARS-CoV-2 transmission, and changes in personal behaviors suppressed influenza activity in South Korea. NPI timing and intensity were associated with decreases in influenza activity during the COVID-19 pandemic. The imperative of comprehensive management strategies to control the spread of influenza remains evident, particularly considering the high rates of mortality and hospitalization observed among high-risk groups due to influenza. Our finding provide insight into the effectiveness of NPIs against IFV and we acknowledge the necessity of further studies to evaluate the effectiveness of different NPIs, some of which are socioeconomically costly and may not be feasible, for the control of influenza. In summary, our findings contribute to an enhanced understanding of the intricate interplay between NPIs and influenza activity during the COVID-19 pandemic. The effectiveness of NPIs in suppressing IFV underscores their potential significance as a tool for public health management. As we navigate the challenges posed by infectious diseases, including influenza, a continued exploration of NPI effectiveness is paramount to inform evidence-based strategies for disease control.

### Supplementary Information

**Additional file 1: Table S1.** Social-distancing levels by period (2020–2021). **Table S2.** Analysis of influenza epidemics based on clinical surveillance data. **Table S3.** Selected SARIMA models. **Table S4.** Parameter of Selected SARIMA models. **Table S5.** Accuracy of Selected SARIMA models. **Figure S1.** Genetic analysis of influenza viruses in the Korea Influenza and Respiratory Viruses Surveillance System. **Figure S2.** Influenza-like illness (ILI) rate and social-distancing (SD) level in South Korea. **Figure S3.** Fitting result of selected SARIMA model.

## Data Availability

The datasets generated and analyzed in this study are available from the corresponding author upon reasonable request.

## Abbreviations

IFV: Influenza virus
IFV A: Influenza virus A
IFV B: Influenza virus B
ILI: Influenza-like illness
NPI: Non-pharmaceutical interventions
