# Supplementary material for: Analysis of the effectiveness of non-pharmaceutical interventions on influenza during the Coronavirus disease 2019 pandemic by time-series forecasting
Source: BMC Infect Dis. 2023 Oct 24;23:717. doi: 10.1186/s12879-023-08640-y (PMC10594831; doi:10.1186/s12879-023-08640-y)
Supplement: Supplementary file 1 — Additional file 1: Table S1. Social-distancing levels by period (2020–2021). Table S2. Analysis of influenza epidemics based on clinical surveillance data. Table S3. Selected SARIMA models. Table S4. Parameter of Selected SARIMA models. Table S5. Accuracy of Selected SARIMA models. Figure S1. Genetic analysis of influenza viruses in the Korea Influenza and Respiratory Viruses Surveillance System. Figure S2. Influenza-like illness (ILI) rate and social-distancing (SD) level in South Korea. Figure S3. Fitting result of selected SARIMA model. [file 12879_2023_8640_MOESM1_ESM.docx]

Table S1. Social-distancing levels by period (2020–2021).

| Period | Week | Social distancing intensity* | Social distancing level |
| --- | --- | --- | --- |
| 2020.01.20. | W4 | Weak | First imported COVID-19 case confirmed in South Korea  Alert level raised to yellow |
| 2020.02.23. | W9 | Weak | Alert level raised to red |
| 2020.02.29.~03.21 | W9~W12 | Weak | Social distancing^1)^ |
| 2020.03.11. | W11 | Weak | WHO declared COVID-19 a pandemic |
| 2020.03.22.~04.19 | W13~W16 | Moderate | Enhanced social distancing^1)^ |
| 2020.04.20.~05.05 | W17~W18 | Weak | Relaxed social distancing^1)^ |
| 2020.05.06.~08.18. | W19~W33 | Weak | Distancing in daily life^1)^ and Level 1 social distancing^2)^ |
| 2020.08.19.~8.29. | W34~W35 | Moderate | Level 2 social distancing^2)3)^ |
| 2020.08.30.~9.13. | W36~W37 | Moderate | Level 2.5 social distancing |
| 2020.09.14.~10.11. | W38~W41 | Moderate | Level 2 social distancing  * 9.28.~10.10.: Holiday special quarantine: |
| 2020.10.12.~11.6. | W42~W45 | Weak | Level 1 social distancing |
| 2020.11.07.~11.18. | W46~W47 | Weak | Level 1 social distancing^4)^ |
| 2020.11.19.~11.23. | W47~W48 | Strong | Level 1.5 social distancing (special quarantine for the national university entrance exam) |
| 2020.11.24.~11.30. | W48~W49 | Very Strong | Level 2 social distancing |
| 2020.12.01.~12.07. | W49 | Very strong | Level 2+a social distancing |
| 2020.12.08.~21.02.14. | W50~W6 | Very strong | Level 2.5 social distancing  *Private gathering restricted |
| 2021.02.15.~07.11 | W7~W27 | Strong | Level 2 social distancing |
| 2021.07.12.~10.31. | W28~W43 | Very strong | Level 4 social distancing^5)^  *9.13.~9.26.: Holiday special quarantine |
| 2021.11.01.~11.28. | W44~W47 | Weak | Step-by step daily recovery |
| 2021.11.29.~12.31. | W48~W52 | Very strong | Special quarantine measure |

^1)^ Social distancing policy divided into three levels

^2)^ 2020.06.28: Social distancing policy reformed to three levels (Level 1, 2, and 3)

^3)^ Only metropolitan areas

^4)^ 2020.11.07: Social distancing altered to five levels (Levels 1, 1.5, 2, 2.5, and 3)

^5)^ 2021.07.12.: Social distancing altered to four levels (Levels 1, 2, 3, and 4)

* The social distancing intensity was classified into four levels by the researchers based on social distancing level

Table S2. Analysis of influenza epidemics based on clinical surveillance data.

| Season | ILI baseline | Flu advisory  issued date (ILI) | Flu advisory  lifted date (ILI) | Duration of epidemic | Peak point (ILI) | |
| --- | --- | --- | --- | --- | --- | --- |
|  |  |  |  |  | 1st peak | 2nd peak |
| 2013–2014 | 12.1 | 14.1.2. (15.3) | 14.5.1. (6.4) | 15 weeks | 64.3 (W7) | - |
| 2014–2015 | 12.2 | 15.1.22. (14.0) | 15.5.21. (6.2) | 17 weeks | 45.5 (W8) | 35.6 (W12) |
| 2015–2016 | 11.3 | 16.1.14. (12.3) | 16.5.27. (6.0) | 17 weeks | 53.8 (W7) | 32 (W14) |
| 2016–2017 | 8.9 | 16.12.8. (13.5) | 17.6.2. (6.7) | 26 weeks | 86.2 (W52) | 16.7 (W14) |
| 2017–2018 | 6.6 | 17.12.1. (7.7) | 18.5.25. (6.0) | 25 weeks | 72.1 (W1) | - |
| 2018–2019 | 6.3 | 18.11.16. (7.8) | 19.6.21. (4.7) | 32 weeks | 73.3 (W52) | 44.2 (W16) |
| 2019–2020 | 5.9 | 19.11.15. (7.0) | 20.3.27. (3.2) | 20 weeks | 49.8 (W52) | - |
| 2020–2021 | 5.8 | not issued | - | - | - | - |
| 2021–2022 | 5.8 | not issued | - | - | - | - |

ILI, influenza-like illness

Table S3. Selected SARIMA models.

| Variable | Selected model | AICc | MASE |
| --- | --- | --- | --- |
| ILI cases/1000 outpatient | ARIMA(2,0,2)(1,1,0)[52] | 1838.535 | 0.302 |
| Influenza (all) positivity rate | ARIMA(2,0,3)(0,1,1)[52] | 1859.892 | 0.308 |
| Influenza A positivity rate | ARIMA(2,0,1)(1,1,0)[52] | 1738.978 | 0.390 |

AICc, corrected Akaike’s Information Criterion; ILI, influenza-like illness

Table S4. Parameter of Selected SARIMA models.

| Influenza (all) positivity rate | | AR(1) | | | AR(2) | | | | MA(1) | | |
| --- | --- | --- | --- | --- | --- | --- | --- | --- | --- | --- | --- |
|  | Coefficients | 1.4009 | | | -0.5495 | | | | -0.4172 | | |
|  | Standard Error | 0.1179 | | | 0.105 | | | | 0.124 | | |
|  |  | MA(2) | | | MA(3) | | | | SMA(1) | | |
|  | Coefficients | 0.2238 | | | 0.2434 | | | | -0.5601 | | |
|  | Standard Error | 0.0622 | | | 0.0846 | | | | 0.0703 | | |
|  |  | AIC=1859.53 | | | | | | | | | |
|  |  | AICc=1859.89 | | | | | | | | | |
|  |  | BIC=1885.75 | | | | | | | | | |
| Influenza A positivity rate | | AR(1) | | AR(2) | | | MA(1) | | | SAR(1) | |
|  | Coefficients | 1.7146 | | -0.8141 | | | -0.665 | | | -0.4458 | |
|  | Standard Error | 0.0554 | | 0.0467 | | | 0.0809 | | | 0.054 | |
|  |  | AIC=1738.78 | | | | | | | | | |
|  |  | AICc=1738.98 | | | | | | | | | |
|  |  | BIC=1757.51 | | | | | | | | | |
| ILI Cases/1000 outpatients | | AR(1) | AR(2) | | | MA(1) | | MA(2) | | | SAR(1) |
|  | Coefficients | 1.1234 | -0.3338 | | | 0.2699 | | 0.2312 | | | -0.4366 |
|  | Standard Error | 0.1584 | 0.1385 | | | 0.16 | | 0.0915 | | | 0.0566 |
|  |  | AIC=1838.26 | | | | | | | | | |
|  |  | AICc=1838.54 | | | | | | | | | |
|  |  | BIC=1860.74 | | | | | | | | | |

Table S5. Accuracy of Selected SARIMA models.

| Variables | Selected Model | RMSSE | MAE | MASE | MAPE |
| --- | --- | --- | --- | --- | --- |
| ILI Cases/1000 outpatients | (2,0,2)(1,1,0)[52] | 0.309 | 2.08 | 0.302 | 0.341 |
| Influenza (all) positivity rate | (2,0,3)(0,1,1)[52] | 0.313 | 2.21 | 0.308 | 55.18 |
| Influenza A positivity rate | (2,0,1)(1,1,0)[52] | 0.370 | 1.73 | 0.390 | 186.1 |

^1)^ During peak season* of IFV: ILI Cases/1000 outpatients MAPE: 0.550, Influenza (all) positivity rate: 0.908, Influenza A positivity rate: 0.510

^2)^ During peak season of IFV A: ILI Cases/1000 outpatients MAPE: 0.504, Influenza (all) positivity rate: 1.086, Influenza A positivity rate: 0.334

*Peak season is defined as the period during which the positivity rate exceeds 10%


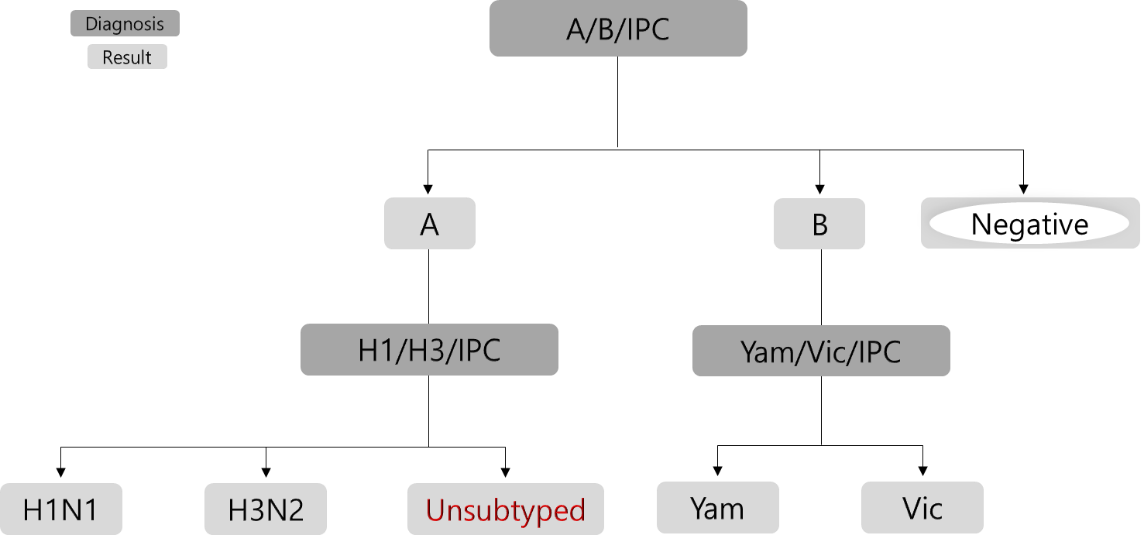


Figure S1. Genetic analysis of influenza viruses in the Korea Influenza and Respiratory Viruses Surveillance System.


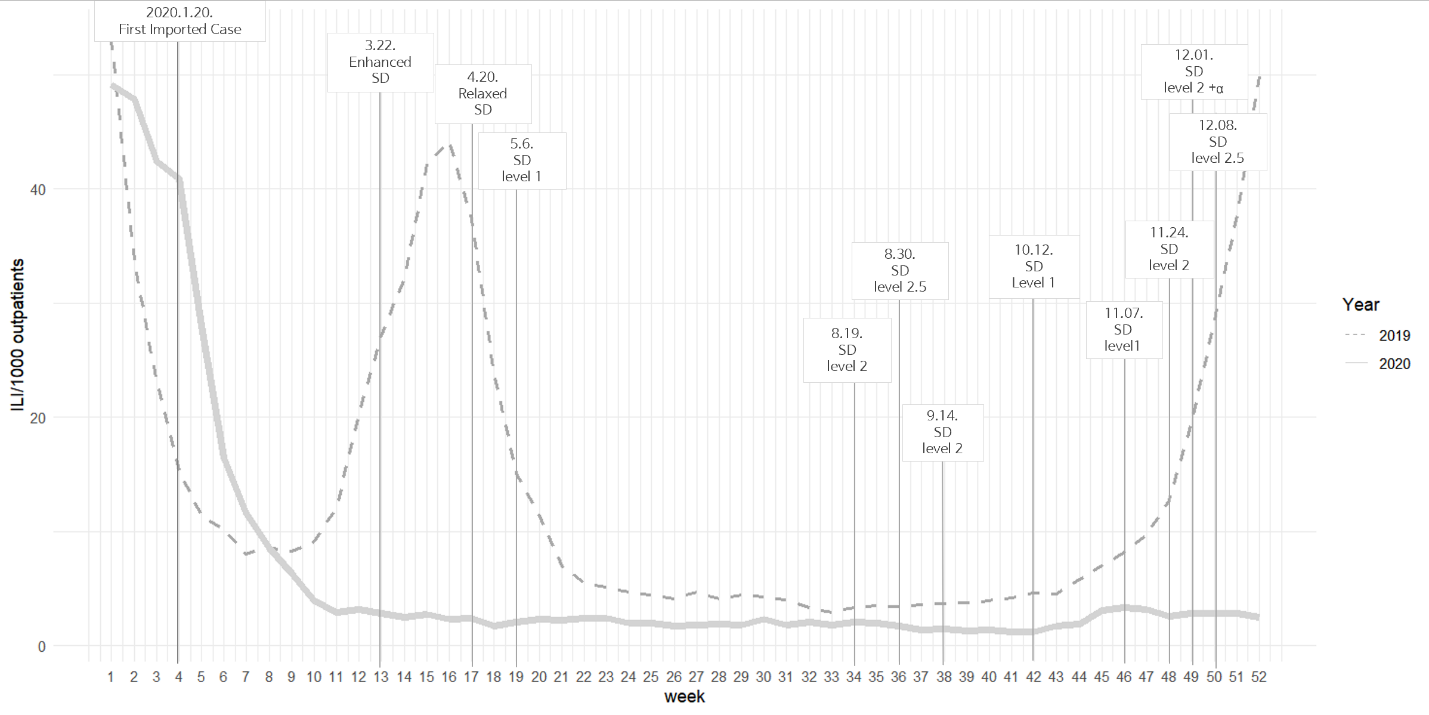


Figure S2. Influenza-like illness (ILI) rate and social-distancing (SD) level in South Korea.

| a) | 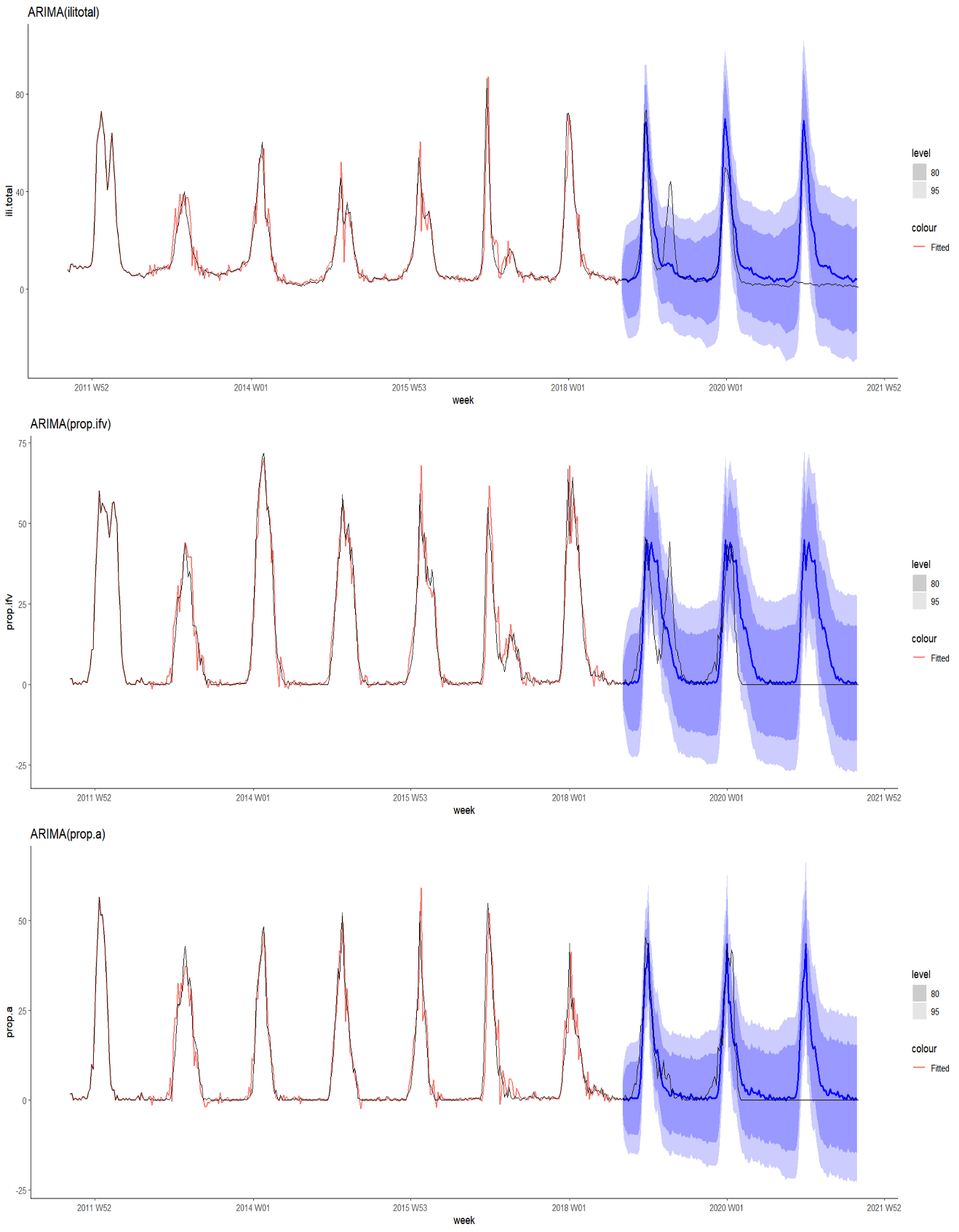 |
| --- | --- |
| b) |  |
| c) |  |

Figure S3. Fitting result of selected SARIMA model.

*Red line indicates fitted value, blue line indicates predicted and black line is observed

a)Forecast of ILI/1000 outpatients

b) Forecast of IFV positive rate

c) Forecast of IFV A positive rate
